# Identifying the origin of nitrous oxide dissolved in deep ocean by concentration and isotopocule analyses

**DOI:** 10.1038/s41598-019-44224-0

**Published:** 2019-05-24

**Authors:** Sakae Toyoda, Osamu Yoshida, Hiroaki Yamagishi, Ayako Fujii, Naohiro Yoshida, Shuichi Watanabe

**Affiliations:** 1 Department of Chemical Science and Engineering, School of Materials and Chemical Technology, Tokyo Institute of Technology, Yokohama, Japan; 2 College of Agriculture, Food and Environment Sciences, Rakuno Gakuen University, Ebetsu, Hokkaido Japan; 3 Interdisciplinary Graduate School of Science and Engineering, Tokyo Institute of Technology, Yokohama, Japan; 4 Earth-Life Science Institute, Tokyo Institute of Technology, Tokyo, Japan; 5 Mutsu Institute for Oceanography, Japan Agency for Marine-Earth Science and Technology, Mutsu, Aomori Japan; 6 Present Address: Environmental Health Department, Ministry of the Environment, Tokyo, Japan; 7 Present Address: Tokyo University of Agriculture, Tokyo, Japan

**Keywords:** Element cycles, Environmental sciences, Marine biology

## Abstract

Nitrous oxide (N_2_O) contributes to global warming and stratospheric ozone depletion. Although its major sources are regarded as bacterial or archaeal nitrification and denitrification in soil and water, the origins of ubiquitous marine N_2_O maximum at depths of 100–800 m and N_2_O dissolved in deeper seawater have not been identified. We examined N_2_O production processes in the middle and deep sea by analyzing vertical profiles of N_2_O concentration and isotopocule ratios, abundance ratios of molecules substituted with rare stable isotopes ^15^N or ^18^O to common molecules ^14^N^14^N^16^O, in the Atlantic, Pacific, Indian, and Southern oceans. Isotopocule ratios suggest that the N_2_O concentration maxima is generated by *in situ* microbial processes rather than lateral advection or diffusion from biologically active sea areas such as the eastern tropical North Pacific. Major production process is nitrification by ammonia-oxidizing archaea (AOA) in the North Pacific although other processes such as bacterial nitrification/denitrification and nitrifier-denitrification also significantly contribute in the equatorial Pacific, eastern South Pacific, Southern Ocean/southeastern Indian Ocean, and tropical South Atlantic. Concentrations of N_2_O below 2000 m show significant correlation with the water mass age, which supports an earlier report suggesting production of N_2_O during deep water circulation. Furthermore, the isotopocule ratios suggest that AOA produce N_2_O in deep waters. These facts indicate that AOA have a more important role in marine N_2_O production than bacteria and that change in global deep water circulation could affect concentration and isotopocule ratios of atmospheric N_2_O in a millennium time scale.

## Introduction

The oceans are estimated as the third largest source of atmospheric N_2_O after natural soil and agriculture^1^. Oceans are also a large reservoir of this greenhouse and ozone-depleting gas because N_2_O has seawater solubility as high as CO_2_ ^2^. Moreover, oversaturation of N_2_O is often found at depths below the surface layer (<ca. 100 m depth)^3^. In many ocean areas, the N_2_O concentration increases with depth and shows a maximum at 100–800 m depth. The highest concentration is found in the eastern tropical North/South Pacific (ETNP/ETSP)^4^. Based on these facts, it has been proposed that N_2_O produced in the ETNP/ETSP, where biological activities are very high because of upwelling of nutrient rich water, is globally transported by isopycnal movement of seawater^4^. However, one earlier report describes that N_2_O concentrations in the water deeper than 2000 m show positive correlation with the age of the water with respect to global circulation^5^, suggesting that N_2_O production might also occur in the deep water. Although deep waters do not contact with the atmosphere, such N_2_O oversaturation is expected to affect ocean-to-atmosphere flux of N_2_O on a longer time scale by diffusive transport or by change in global ocean circulation. Nevertheless, the origin and production mechanisms of the N_2_O maximum and the N_2_O in deep water remain uncertain.

Isotopocule ratios (δ^15^N, δ^18^O, and SP, ^15^N-site preference in NNO molecule) of N_2_O are useful parameters to identify the origin and production–consumption processes of N_2_O because they depend on the isotopic ratios in precursors and isotope effects of chemical or biochemical reactions^6^. Reportedly, the magnitudes of isotope effects on nitrogen during N_2_O production as byproduct of nitrification by ammonia oxidizing bacteria (AOB)^7,8^ and ammonia oxidizing archaea (AOA)^9,10^ differ. Moreover, SP values of N_2_O produced by AOB via hydroxylamine (NH_2_OH)^9,11^ and by AOA^9,10,12^ are distinct from the values of N_2_O produced by AOB via nitrite (NO_2_^−^)^8,11,13,14^ or by denitrification^11,15^ (Supplementary Fig. S1, Table S2). When N_2_O is partially reduced to N_2_ in denitrification, all isotopocule ratios increase^16–20^. Although the isotope effect during N_2_O reduction varies among bacterial species or pure culture and community incubations, consistent relations have been found between isotope effects on N, O, and SP^16^.

**Figure 1:**
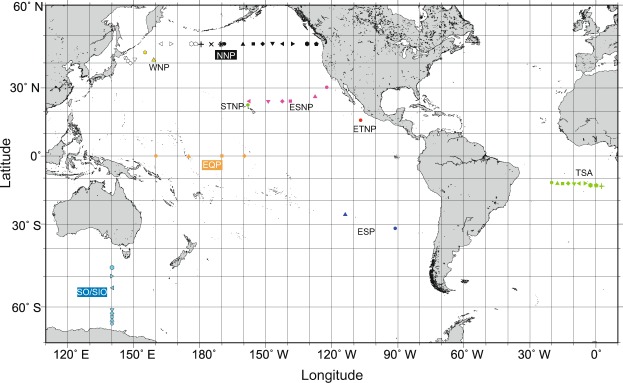
Map showing seawater sampling stations. In this work, observations in the northern North Pacific (NNP), equatorial Pacific (EQP), and Southern Ocean/southeastern Indian Ocean (SO/SIO) were conducted. Data in other regions were compiled from previous studies. See also Table S1. Each symbol corresponds to the symbol presented in Figs 2 and 3.

Several earlier studies have analyzed vertical profiles of concentration and isotopocule ratios of N_2_O in the ocean and have investigated production–consumption processes specific to the depths or sites^17,21–25^. Nevertheless, it remains unclear whether N_2_O is produced *in situ* or transported from other regions because existing data do not cover the wide range of oceanic setting such as circulation age of deep seawater. This report describes new isotopocule analyses of N_2_O in the northern North Pacific (NNP), equatorial Pacific (EQP), and Southern Ocean and southeastern Indian Ocean (SO/SIO) where younger, medium, and older water respectively exist in deep layers (Fig. 1). The respective results are compared with existing observations to ascertain the origin of N_2_O from the perspective of global deep water circulation.

## Vertical Profiles of Dissolved N_2_O

**Figure 2:**
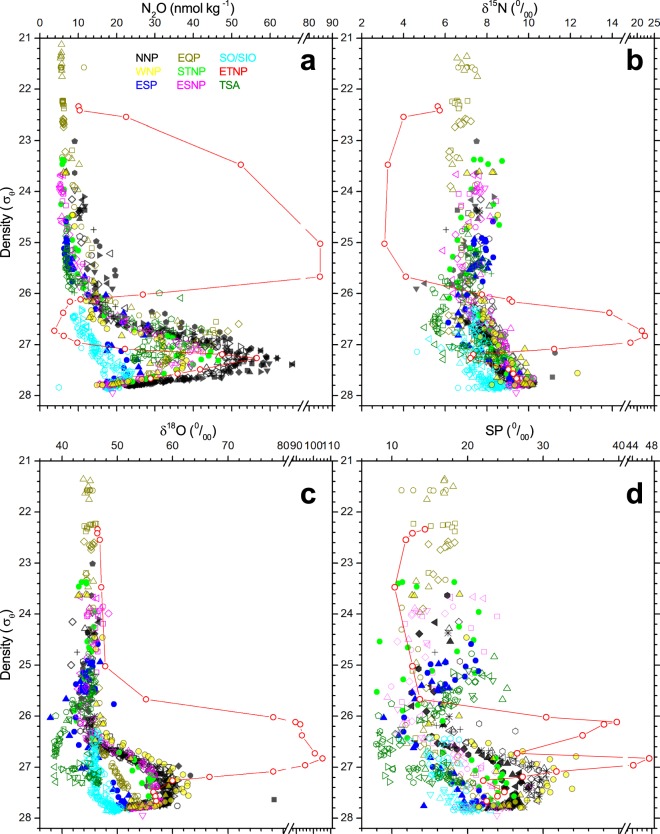
Vertical distribution of concentrations and isotopocule ratios of N_2_O in nine oceanic regions. **(a**–**d)** Concentration (**a**), δ^15^N^bulk^ (**b**), δ^18^O (**c**), and *SP* (**d**). Symbol colors represent regions. Vertical axis shows potential density anomaly instead of depth, in order to compare the N_2_O profiles obtained from regions with different physical structures. When samples were collected at several stations in a region, the symbol type was changed accordingly: NNP, northern North Pacific; EQP, equatorial Pacific; SO/SIO, Southern Ocean/southeastern Indian Ocean; WNP, western North Pacific; STNP, subtropical North Pacific; ETNP, eastern tropical North Pacific; ESP, eastern South Pacific; ESNP, eastern subtropical North Pacific; TSA, tropical South Atlantic. See Table S1 for data sources.

In the surface layer, concentrations of N_2_O measured in the eight regions except the eastern tropical North Pacific (ETNP) are as low as those expected under the dissolution equilibrium between atmosphere and seawater (6–8 nmol kg^−1^). However, they increase with depth and reach a maximum (25–65 nmol kg^−1^) (Fig. 2a). The depth of the maximum is 100–400 m in the tropical South Atlantic (TSA), SO, and EQP, and 400–1500 m in other regions. Those values correspond to almost identical seawater density (potential density anomaly, *σ*_*θ*_, of about 27) (Supplementary Table S1). Below the maximum, the N_2_O concentration decreases with depth. It reaches 15–40 nmol kg^−1^ in the deep layer (>2000 m depth or *σ*_*θ*_ = 27.7–27.8). In the ETNP, two concentration maxima exist at 60 m (*σ*_*θ*_ = 25.0) and 800 m (*σ*_*θ*_ = 27.3)^17^.

Each of the three independent isotopocule ratios shows a unique profile that is different from that of concentration (Fig. 2b–d). The bulk (or average) nitrogen isotope ratio (δ^15^N^bulk^) in the eight regions except ETNP shows no vertical gradient from the surface to the depth corresponding to *σ*_*θ*_ ≅ 25. Then it decreases slightly with depth, showing a minimum value at *σ*_*θ*_ ≅ 26. Below the small minimum, it increases monotonically with depth and reaches its maximum (9–10‰) at the bottom layer. In the ETNP, it shows a minimum and a maximum respectively corresponding to the shallow and deep concentration maxima. The oxygen isotope ratio (δ^18^O) and ^15^N-site preference (SP) also show almost constant values from the surface to the depth above the concentration maximum (*σ*_*θ*_ ≅ 26) in the eight regions. In deeper water, however, their vertical profiles are markedly different among oceanic regions. Although δ^18^O and SP respectively show a parallel increase and decrease with concentration in the North Pacific stations, they exhibit a monotonic increase in EQP, SO/SIO, and ESP. In TSA, they show minima at the concentration maximum.

## Origin of N_2_O at Concentration Maximum

**Figure 3:**
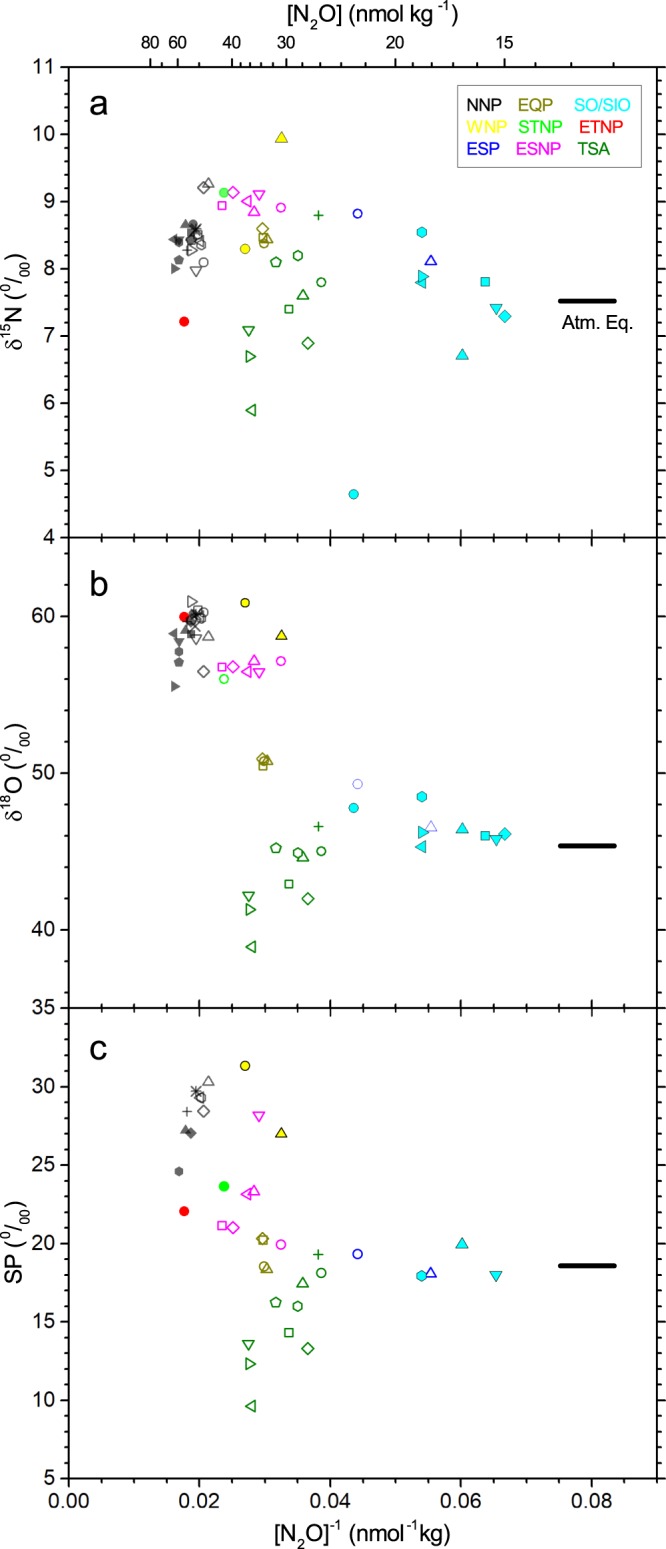
Comparison of isotopocule ratios of N_2_O at concentration maximum in nine oceanic regions. (**a**–**c**) Relations between inverse concentration and δ^15^N^bulk^ (**a**), δ^18^O (**b**), and *SP* (**c**). Colors and types of symbols are as shown in Fig. 2. Horizontal solid lines show values of dissolved N_2_O when the seawater is equilibrated with the atmosphere.

**Figure 4:**
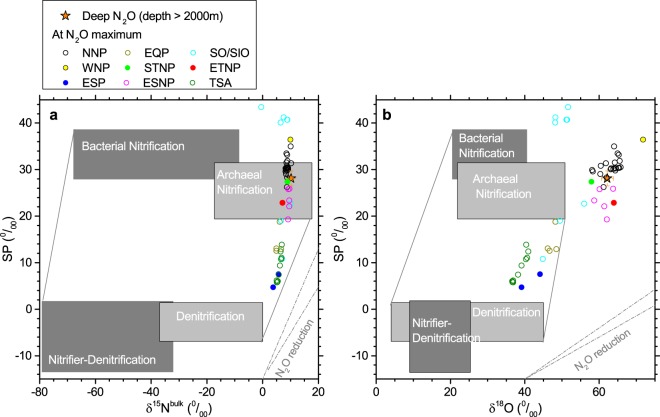
Comparison of isotopocule ratio of the excess N_2_O produced at concentration maximum (circles) and deeper seawater (orange star). (**a,b**) Relation between δ^15^N^bulk^ and *SP* (**a**) and the relation between δ^18^O and *SP* (**b**). Rectangles respectively show ranges expected when N_2_O is produced by the indicated microbial processes (See Supplementary Information). Broken lines show range of the slope expected for increase in isotopocule ratios during partial reduction by denitrification.

Isotopic signatures of N_2_O at density level of *σ*_*θ*_ = 27.3 which corresponds to concentration maximum in ETNP show systematic difference between oceanic regions (Fig. 3). For example, in spite of similarity in water mass property (temperature and salinity, data not shown) between NNP and WNP and between EQP and ETNP, they are distinguished each other. Variations of isotopocule ratios are caused by (1) mixing of N_2_O with different isotopic signatures such as N_2_O produced *in situ*, advected from other regions, and dissolved atmospheric N_2_O or (2) decomposition of N_2_O during which the remaining N_2_O is isotopically fractionated^6^. Based on a global distribution of N_2_O concentration, it has been proposed that N_2_O produced in the eastern tropical Pacific is exported to other regions in the Pacific by lateral or isopycnal advection^4^. If this is the case, and if we consider mixing of two endmembers, namely, N_2_O produced in the ETNP and background N_2_O from the atmosphere, the isotopic data points for the regions outside the ETNP is expected to fall on the mixing line in isotope–reciprocal concentration plot. However, the results show that mixing between N_2_O in the ETNP and N_2_O in the water equilibrated with the atmosphere cannot explain the observed isotopocule ratios in the eight regions (Fig. 3). In other words, the isotopocule ratios for excess N_2_O at N_2_O maxima in the eight regions, as calculated assuming isotopic mass balance between observed and background N_2_O, are distinct from those obtained at ETNP (Fig. 4 and Table S1).

In Fig. 3, N_2_O maxima in NNP could be regarded as an alternative endmember because of its high concentration. However, lateral advection of N_2_O from NNP is also improbable for the following reasons. First, the distribution of data obtained in other regions still cannot be explained consistently by the isopycnal mixing in each panel of Fig. 3. Second, vertical profiles of temperature and salinity at the stations in this work do not show discontinuous feature which is expected if different water mass is advected laterally.

Contribution from partial reduction of N_2_O (the second factor noted above) is also unlikely because all the isotopic signatures should be increased with decreasing concentration and because nitrate reduction, which is the first step of denitrification and prerequisite for N_2_O reduction, is not prominent in the observed oxic water columns. Therefore, we infer that the N_2_O maximum is a result of *in situ* production rather than advection from other regions. Possible production mechanisms are discussed below together with those for deep water N_2_O.

## Origin of N_2_O in Deep Layer

**Figure 5:**
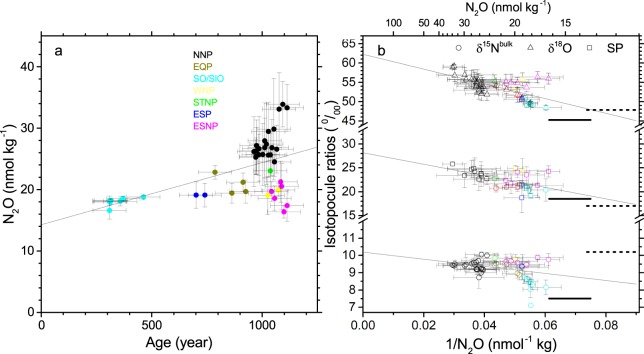
Concentration and isotopocule ratios of N_2_O for deep water (depth >2000 m) in nine oceanic regions and their relation with circulation age of seawater. (**a,b**) Relation between circulation age and N_2_O concentration (**a**) and relation between iverse concentration and isotopocule ratios of N_2_O (**b**). Each symbol denotes the average for the deep water at each station. Error bars for concentration and isotopocule ratios show one standard deviation while those for age are reported estimated error^26^. Solid and broken horizontal lines in panel b respectively show the values expected when the surface water in the subduction region is equilibrated with modern and preindustrial atmosphere^31^. Correlation coefficients of regression lines and probabilities of null hypothesis in ANOVA are as follows. In panel a, *r* = 0.55, *p* = 1.3 × 10^–4^; in panel b, δ^15^N^bulk^, *r* = −0.38, *p* = 1.3 × 10^−2^; δ^18^O, *r* = −0.65, *p* = 2.3 × 10^−6^; *SP*, *r* = ^−^0.56, *p* = 1.4 × 10^−3^.

The concentration of N_2_O averaged for water below 2000 m depth shows a positive correlation with the circulation age of seawater estimated from the ^14^C content^26^ (Fig. 5a). Bange and Andreae found a similar relation by compiling 56 observations from the North/South Atlantic, North/South Indian, and North Pacific oceans^5^. Based on insignificant fluxes estimated for hydrothermal or sediment N_2_O, they concluded that N_2_O is produced in the deep ocean mainly by nitrification. The slope of the regression line in Fig. 5a ((1.0 ± 0.2) × 10^−2^ nmol kg^−1^ yr^−1^) is comparable to their reported value of (5.7 ± 1.0) × 10^−3^ nmol L^−1^ yr^−1^ and the y-intercept (14.3 ± 2.2 nmol kg^−1^) agrees with the earlier report (13.1 nmol kg^−1^)^5^. Here we show that isotopocule ratios also increase with the circulation age. When plotted against inverse N_2_O concentration, they are distributed on a line that passes ranges of surface water that is saturated with the atmosphere (Fig. 5b). This confirms that N_2_O is added during the circulation of deep seawater after losing contact with the atmosphere, and the following isotopocule ratios of the produced N_2_O are obtained as y-intercepts of the regression lines: 10.2 ± 0.4‰, 62.2 ± 1.6‰, and 28.1 ± 1.6‰, respectively, for δ^15^N^bulk^, δ^18^O, and SP.

A closer look at Fig. 5a and Supplementary Table S1 reveals systematic difference in N_2_O concentration between NNP, STNP, WNP, and ESNP. Since it has been suggested that deep water circulates from the South Pacific to these north Pacific regions via different routes^27,28^, N_2_O production rate during the circulation might vary with the pathways. For instance, dissolved oxygen concentration in the deep layer is lowest in NNP^29^, which could explain the higher N_2_O concentration in NNP than other North Pacific regions because N_2_O production is enhanced under low oxygen condition^12,30^. Additional factor that could partly explain the positive deviations of NNP data from the fitted line is underestimation of age for the NNP waters (Matsumoto, personal communication).

In consideration of millennium scale of deep water circulation, concentration and isotopic signature of N_2_O initially existed in the water mass before subduction do not equal to the present values. Solid and broken horizontal lines in Fig. 5b respectively show the values for the surface water in the subduction region (off Greenland and off Antarctica) which is equilibrated with modern and preindustrial atmosphere^31^. The negative deviation of the SO/SIO data might indicate that relatively younger water mass in this region is affected by the mixing of modern surface water during its advection.

## Production Mechanisms of N_2_O in Concentration Maximum and Deep Layer

We compare the isotopocule ratio values of excess N_2_O at concentration maximum and accumulated N_2_O during deep water circulation with those values reported or estimated for currently known biological processes of oceanic N_2_O (Fig. 4, Supplementary Table S4). The ranges of δ^15^N^bulk^ and SP values for the N_2_O maximum at most of the stations in the North Pacific and the values for deep N_2_O overlap with that of N_2_O produced by archaeal nitrification conducted by ammonia-oxidizing archaea (AOA)^9,10,12^ (Fig. 4a). At several stations in the North Pacific and SO/SIO, SP values of N_2_O at maximum concentration are larger than the range of AOA-derived N_2_O, suggesting additional contribution from bacterial nitrification. In the Atlantic and at several stations in the equatorial/South Pacific and SO/SIO, N_2_O maxima are accompanied by lower SP values, which indicates significant role of nitrifier-denitrification/denitrification. In contrast, δ^18^O values obtained for both N_2_O maximum and deep water N_2_O are higher than that of AOA by 5–20‰ (Fig. 4b). If N_2_O produced by AOA or bacterial processes is further reduced by denitrification, δ^18^O of residual N_2_O increases due to isotope fractionation^16–20^. However, the parallel increases in δ^15^N^bulk^ and *SP* expected for N_2_O reduction (slope of broken lines in Fig. 4) are not prominent especially for δ^15^N^bulk^ and it is unlikely that N_2_O reduction occurs in oxic seawater because N_2_O reduction has been found only in anoxic environment such as ETNP or Arabian Sea^17,32^. Therefore, the discrepancy of δ^18^O might be explained by variation of oxygen isotope effects during N_2_O production by AOA. This should be tested using further laboratory studies with several species of AOA. Reported oxygen isotope fractionation during the incorporation of O_2_ into N_2_O is −2.2 to 13.2‰ in two marine and five soil archaeal species/isolates^9,10^. Those figures might become larger if the reaction proceeds with the equilibrium step because O-isotope exchange equilibrium fractionation between N_2_O and O_2_ is calculated theoretically as about 20‰ at 0–10 °C^33^.

Several reports have described the dominance of AOA in nitrification in the ocean. Archaeal *amoA* gene abundance was positively correlated with potential NH_3_ oxidation rate and N_2_O concentration in the upper oxycline of the eastern tropical South Pacific^34^. In the oxygen minimum zone (OMZ) of the eastern tropical North Atlantic, comparable patterns of abundance and expression of archaeal *amoA* genes and N_2_O co-occurred^12^. Our results suggest that AOA play a major role not only in the OMZ of tropical ocean but also in the OMZ in temperate and subarctic ocean areas and deeper waters. The average rate of N_2_O production during deep water circulation estimated from the relation between N_2_O concentration and circulation age of seawater is 28 f mol L^−1^ d^−1^, which is comparable to the rate determined experimentally with pure culture of marine AOA *Nitrosopumilus maritimus* SCM1 (4.4 × 10^−4^–2.4 × 10^−2^ f mol d^−1^ cell^−1^ under oxygen concentrations of 203–34 μmol L^−1^)^12^ if one assumes cell density of AOA in deep waters as 10^4^ cells L^−1^.

Our results suggest that ubiquitous N_2_O maximum in the middle layer in the North Pacific is formed by *in situ* production (mainly by AOA) rather than advection from the eastern tropical Pacific and that N_2_O is also produced by AOA and accumulated during the global deep water circulation. If one simply assumes that the N_2_O dissolved in the oldest deep water in NNP (34 nmol kg^−1^) is ultimately released to the atmosphere by the circulation driven by seawater subduction in the polar regions (3 × 10^7^ m^3^ s^−1^)^35^, magnitude of this deep N_2_O source is estimated at up to 1 Tg N yr^−1^ compared to the estimated surface oceanic source of 3.8 Tg N yr^−1^ ^1^ which seems to be based on limited surface observations. This implies that change in global deep water circulation could affect concentration and isotopocule ratios of atmospheric N_2_O in a millennium time scale.

## Methods

Seawater samples were collected at 21 stations in the North Pacific (40–47°N, 146°E–127°W) during July–August 2007 and 4 stations in the equatorial Pacific (0°N, 160°E–158°W) in January 2003 during the MR07-04 and MR02-K06 cruises, respectively, of R/V *Mirai* (JAMSTEC, Japan) and at 8 stations in the Southern Ocean/southeastern Indian Ocean (47–65°S, 140°E) in Jan 2002 during the KH01-3 cruise of R/V *Hakuho-maru* (JAMSTEC, Japan). At each station, samples were taken at 15–28 depths in the range of 0–6000 m using 12 L Niskin bottles mounted on a conductivity-temperature-depth Rosette sampler. Each sample was collected in a 230 mL glass vial followed by addition by 1 mL saturated HgCl_2_ solution for sterilization and by sealing with a butyl rubber stopper. Each was then preserved at 4 °C in the dark.

Concentration and isotopocule ratios of N_2_O were measured using a gas chromatograph – isotope ratio mass spectrometer (GC-IRMS) equipped with a gas extraction and cryogenic concentration unit as described elsewhere^17,36^. Isotopocule ratios ^15^*R*^α^ (=[^14^N^15^N^16^O]/[^14^N^14^N^16^O]), ^15^*R*^β^(=[^15^N^14^N^16^O]/[^14^N^14^N^16^O]), and ^18^*R* (=[^14^N^14^N^18^O]/[^14^N^14^N^16^O]) are expressed as delta values as defined below^37^.

1 $$\delta X=(R\,-\,{R}_{{\rm{standard}}})/{R}_{{\rm{standard}}}$$

Therein, X denotes ^15^N^α^, ^15^N^β^, or ^18^O; *R* represents ^15^*R*^α^, ^15^*R*^β^, or ^18^*R*; *R*_standard_ means [^15^N^14^N]/[^14^N^14^N] of atmospheric N_2_ or [H_2_^18^O]/[H_2_^16^O] of Vienna Standard Mean Ocean Water. In this report, we use δ value for bulk N and ^15^N-site preference instead of δ^15^N^α^ and δ^15^N^β^:

2 $${\delta }^{15}{{\rm{N}}}^{{\rm{bulk}}}=({\delta }^{15}{{\rm{N}}}^{{\rm{\alpha }}}+{\delta }^{15}{{\rm{N}}}^{{\rm{\beta }}})/2,$$

3 $$SP={\delta }^{15}{{\rm{N}}}^{{\rm{\alpha }}}-{\delta }^{15}{{\rm{N}}}^{{\rm{\beta }}}.$$

The typical analytical precision (1*σ*) is 1% for concentration, and 0.2‰, 0.4‰, and 0.9‰, respectively, for δ^15^N^bulk^, δ^18^O, and SP.

Isotopocule ratios of excess N_2_O at its maximum and those of N_2_O produced *in situ* during deep water circulation were estimated by assuming the mixing of two end members.

4 $${C}_{{\rm{obs}}}={C}_{{\rm{pro}}}+{C}_{{\rm{atm}}}$$

5 $${\delta }_{{\rm{obs}}}{C}_{{\rm{obs}}}={\delta }_{{\rm{pro}}}{C}_{{\rm{pro}}}+{\delta }_{{\rm{atm}}}{C}_{{\rm{atm}}}$$

Equations (4) and (5) respectively describe the mass balance of light and heavy N_2_O molecules; *C*_obs_, *C*_pro_, *C*_atm_ respectively denote the observed, produced, and atmospheric equilibrium concentrations; δ_obs_, δ_pro_, and δ_atm_ are the respective isotopocule ratios. By eliminating *C*_pro_ from Eqs (4) and (5), we obtain the following:

6 $${\delta }_{{\rm{pro}}}=({\delta }_{{\rm{obs}}}{C}_{{\rm{obs}}}\,-\,{\delta }_{{\rm{atm}}}{C}_{{\rm{atm}}})/({C}_{{\rm{obs}}}\,-\,{C}_{{\rm{atm}}})$$

The circulation age of deep seawater at each observational station was calculated from the objectively mapped circulation ^14^C age below 1500 m in the literature^26^ using “2D estimation” tool of Ocean Data View software^38^.

## Supplementary information

Supplementary information

## Data Availability

Data presented in Fig. 2 are available upon request, and will be archived at http://www.godac.jamstec.go.jp/darwin/e or https://www.jodc.go.jp/jodcweb/index.html.
