# Supplementary material for: Identifying the origin of nitrous oxide dissolved in deep ocean by concentration and isotopocule analyses
Source: Sci Rep. 2019 May 24;9:7790. doi: 10.1038/s41598-019-44224-0 (PMC6534561; doi:10.1038/s41598-019-44224-0)
Supplement: Supplementary file 1 — Supplementary information [file 41598_2019_44224_MOESM1_ESM.docx]

**Supplementary information**

**Identifying the origin of nitrous oxide dissolved in deep ocean by concentration and isotopocule analyses**

Sakae Toyoda^1*^, Osamu Yoshida^2^, Hiroaki Yamagishi^3,6^, Ayako Fujii^3,7^, Naohiro Yoshida^1,4^, Shuichi Watanabe^5^

^1^Department of Chemical Science and Engineering, School of Materials and Chemical Technology, Tokyo Institute of Technology, Yokohama, Japan

^2^College of Agriculture, Food and Environment Sciences, Rakuno Gakuen University, Ebetsu, Hokkaido, Japan

^3^Iinterdisciplinary Graduate School of Science and Engineering, Tokyo Institute of Technology, Yokohama, Japan

^4^Earth-Life Science Institute, Tokyo Institute of Technology, Tokyo, Japan

^5^Mutsu Institute for Oceanography, Japan Agency for Marine-Earth Science and Technology, Mutsu, Aomori, Japan

^6^ Present address: Environmental Health Department, Ministry of the Environment, Tokyo, Japan

^7^ Present address: Tokyo University of Agriculture, Tokyo, Japan

**Estimation of isotopic ranges of N_2_O produced by each microbial process**

Ranges shown as rectangles in Fig. 4 were calculated based on isotopic fractionation (ε values, Table S2) and isotope ratios of substrates of N_2_O (Table S3) in the literature. For example, the range for N_2_O from NH_3_ oxidation via NH_2_OH by nitrifying bacteria (Table S4) was estimated as follows.

δ^15^N_min_ = δ^15^N_NH4min_ + ε(^15^N)_AOBmin_,

δ^15^N_max_ = δ^15^N_NH4max_ + ε(^15^N)_AOBmax_,

δ^18^O_min_ = δ^18^O_O2min_ + ε(^18^O)_AOBmin_,

δ^18^O_max_ = δ^18^O_O2max_ + ε(^18^O)_AOBmax_,

while SP values were same as those in Table S2 assuming that they do not depend on δN values of substrates (Toyoda et al., 2005; Sutka et al., 2006).

**Supplementary Tables and Figures**

Table S1. List of observational data used in this study.

Table S2. Isotopic fractionation or SP values during N_2_O production and consumption by bacteria and archaea

Table S3. Range of nitrogen and oxygen isotope ratios for substrates of N_2_O production.


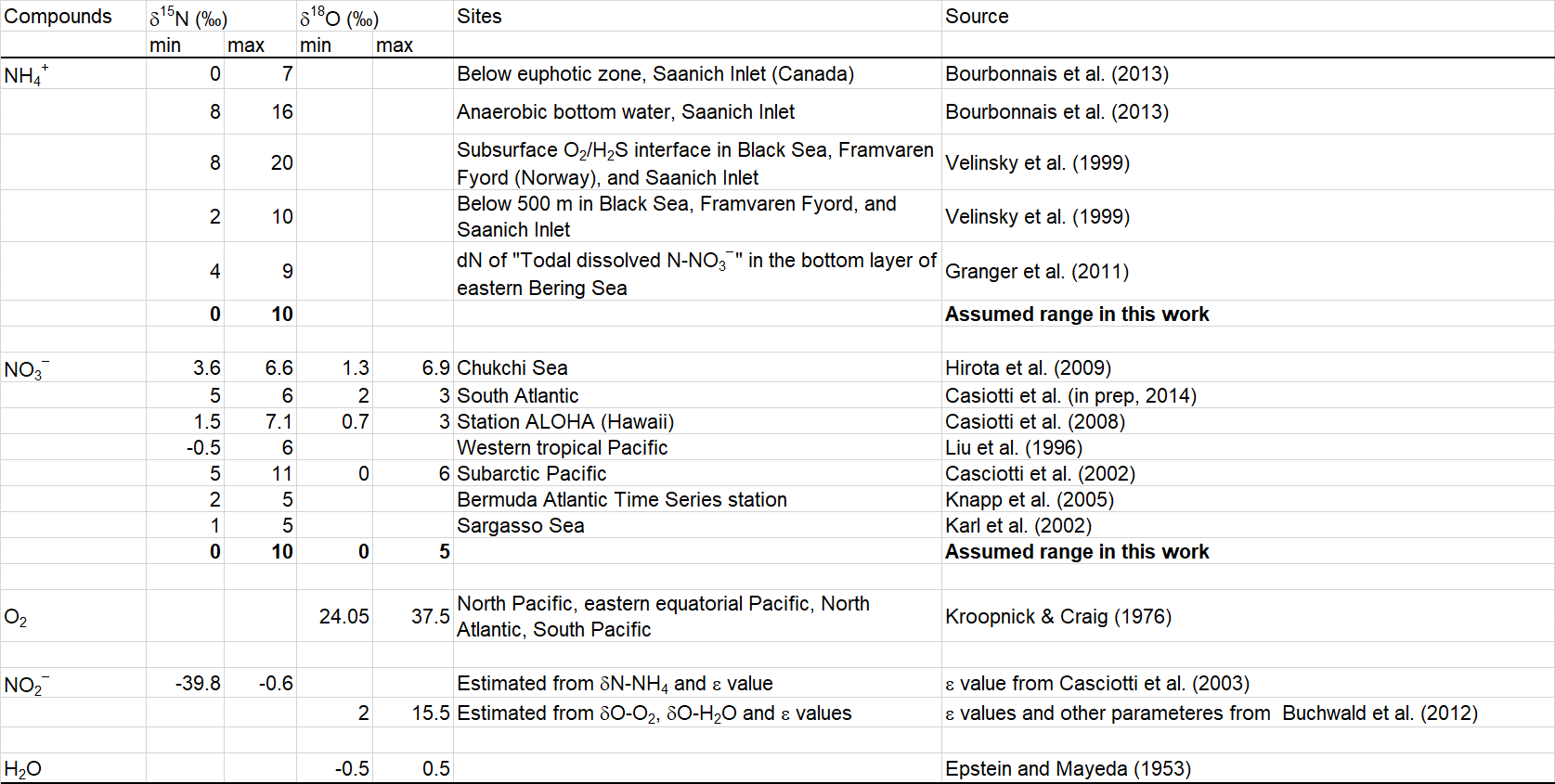


Table S4. Estimated range of isotopocule ratios of N_2_O produced at concentration maximum and in deep ocean.

Fig. S1. Production and consumption processes of N_2_O in the ocean　(Modified from Toyoda et al. 2017).

**References*** *Those listed in the reference section of main text are not listed.

Bourbonnais, A., R. T. Letscher, H. W. Bange, V. Echevin, J. Larkum, J. Mohn, N. Yoshida, and M. A. Altabet (2017), N_2_O production and consumption from stable isotopic and concentration data in the Peruvian coastal upwelling system, *Global Biogeochem. Cycles*, *31*(4), 678–698, doi:10.1002/2016gb005567.

Buchwald, C., A. E. Santoro, M. R. McIlvin, and K. L. Casciotti (2012), Oxygen isotopic composition of nitrate and nitrite produced by nitrifying cocultures and natural marine assemblages, *Limnol. Oceanogr.*, *57*(5), 1361–1375, doi:10.4319/lo.2012.57.5.1361.

Casciotti, K. L., D. M. Sigman, M. G. Hastings, J. K. Böhlke, and A. Hilkert (2002), Measurement of the oxygen isotopic composition of nitrate in seawater and freshwater using the denitrifier method, *Anal. Chem.*, *74*, 4905–4912.

Casciotti, K. L., D. M. Sigman, and B. B. Ward (2003), Linking diversity and stable isotope fractionation in ammonia-oxidizing bacteria, *Geomicrobiology Journal*, *20*, 335–353.

Casciotti, K. L., T. W. Trull, D. M. Glover, and D. Davies (2008), Constraints on nitrogen cycling at the subtropical North Pacific Station ALOHA from isotopic measurements of nitrate and particulate nitrogen, *Deep Sea Res., Part II*, *55*, 1661–1672, doi:10.1016/j.dsr2.2008.04.017.

Epstein, S., and T. Mayeda (1953), Variation of O18 content of waters from natural sources, *Geochim. Cosmochim. Acta*, *4*(5), 213–224, doi:https://doi.org/10.1016/0016-7037(53)90051-9.

Granger, J., M. G. Prokopenko, D. M. Sigman, C. W. Mordy, Z. M. Morse, L. V. Morales, R. N. Sambrotto, and B. Plessen (2011), Coupled nitrification-denitrification in sediment of the eastern Bering Sea shelf leads to15N enrichment of fixed N in shelf waters, *J. Geophys. Res.*, *116*(C11), doi:10.1029/2010jc006751.

Hirota, A., A. Ijiri, D. D. Komatsu, S. B. Ohkubo, F. Nakagawa, and U. Tsunogai (2009), Enrichment of nitrous oxide in the water columns in the area of the Bering and Chukchi Seas, *Marine Chemistry*, *116*, 47–53, doi:10.1016./j.marchem.2009.09.001.

Karl, D., A. Michaels, B. Bergman, D. Capone, E. Carpenter, R. Letelier, F. Lipschultz, H. Paerl, D. M. Sigman, and L. Stal (2002), Dinitrogen fixation in the world’s oceans, *Biogeochemistry*, *57/58*, 47–98.

Knapp, A. N., D. M. Sigman, and F. Lipschultz (2005), N isotopic composition of dissolved organic nitrogen and nitrate at the Bermuda Atlantic Time-series Study site, *Global Biogeochem. Cycles*, *19*(1), GB1018, doi:10.1029/2004gb002320.

Kroopnick, P., and H. Craig (1976), Oxygen isotope fractionation in dissoved oxygen in the deep sea, *Earth and Planetary Science Letters*, *32*, 375-388.

Liu, K. K., M. J. Su, C. R. Hsueh, and G. C. Gong (1996), The nitrogen isotopic composition of nitrate in the Kuroshio Water northeast of Taiwan: evidence for nitrogen fixation as a source of isotopically light nitrate, *Mar. Chem.*, *54*, 273–292.

Velinsky, D. J., and M. Fogel (1991), Isotopic fractionation of dissolved ammonium at the oxygen-hydrogen sulfide interface in anoxic waters, *Geophys. Res. Lett.*, *18*(4), 649–652.

Yoshida, N., A. Hattori, T. Saino, and S. M. E. Wada (1984), ^15^N/^14^N ratio of dissolved N_2_O in the eastern tropical Pacific Ocean, *Nature*, *307*, 442–444.
